# Notes and News

**Published:** 1900-03-03

**Authors:** 


### Notes and News.

### HOSPITAL MEETINGS.

Royal Hospital for Children and Women.—Annual Meeting at three p.m., on Friday, March 2nd, at the Hospital.

Royal Westminster Ophthalmic Hospital, King William Street, Strand.—Annual Meeting at four p.m., on Friday, March 2nd, at the Hospital.

West London Hospital, Hammersmith Road.—Annual Meeting at five p.m., on Monday, March 5th, at the Hospital.

City of London Hospital for Diseases of the Chest.—Annual Meeting at four p.m., on Monday, March 5th, at Gresham College, Basinghall Street.

London Hospital.—Quarterly Court on Wednesday, March 7th.

Chelsea Hospital for Women.—Annual Meeting at half-past three p.m., on Tuesday, March 6th.

Sir Savile Crossley has been elected chairman of the Hospital Saturday Fund for the ensuing year.

The new Isolation Hospital for the Borough of Southampton has been opened by the Mayor. The institution has been built at a cost of £30,000.

The Government have accepted the offer of the yachts "Golden Eagle" and "Sunrise," belonging to Sir Samuel Scott and Mr. Coote, as annexe hospitals to the hospital ship "Nubia."

At a meeting of the Isle of Wight Infirmary and County Hospital, at which Princess Henry of Battenberg presided, it was announced that five cots in the Diamond Jubilee Ward had been endowed with the sum of £1,000 each.

The supply of medical men is of very unequal proportion in European countries. Whilst in Russia the number of medical students who may study at one time at the various Universities is strictly limited, in Hungary fees for instruction in medical subjects are being largely remitted, in order to encourage a larger number of students.

A hospital for infectious diseases has been opened at Braintree, in Essex. It consists of two wards separated by premises for the administration. The wards contain two and three beds respectively. In the keeper's cottage two rooms, each containing four beds, are set apart for emergencies. Small-pox cases will not be admitted. The cost, estimated at about £3,000, will be borne by the Braintree Rural and Urban Councils.

Mr. Treves has had to leave Chieveley Camp for Durban to recruit from a bad attack of dysentery.

The Isolation Hospitals Bill has passed the House of Lords, and is now before the House of Commons.

The plague, which has appeared in New Caledonia, is at present limited to Numea, where the largest number of victims have been amongst the Kanakas and Chinese populations.

Professor Yelverton Pearson, Professor of Materia Medica at Queen's College, Cork, has been appointed Professor of Surgery at that college. He succeeds the late Professor O'Sullivan.

It is suggested that the Professorship of Practice of Physic in the University of Edinburgh, rendered vacant by the death of Sir T. Grainger-Stewart, might attract Professor Osler from Baltimore, if it were offered to him.

Wales has now decided to send a hospital to South Africa for the wounded. A committee has been appointed; contributions have already been received, and can be sent to Professor Hughes, 7, Chester Terrace, Regent's Park, who will supply all particulars.

The British Medical Association fund in aid of the R.A.M.C. in South Africa is making good progress. Numbers of members of the association are collecting in various districts, and contributions may also be sent to the Editor of the *British Medical Journal.*

Alderman Treloar informs us that a successful collection was made for his fund to provide Christmas hampers for crippled children last year. The total receipts were £1,267, of which £1,050 was spent upon hampers, and £95 upon the banquet which was given to crippled children able to attend.

Very many alterations have been made at the Royal South Hants and Southampton Hospital, at which Princess Beatrice opened the new wing recently; indeed, the old building has practically been reconstructed inside. A new staircase, a board room, secretary's office, children's ward, sanitary arrangements, nurses' bedrooms, and numerous other additions and improvements are the result of the alterations made. The new wing consists of a ward for 25 beds and administration block, and the additions also comprise mortuary and isolation buildings. The total cost has been £24,000, exclusive of furnishing.

Since the arrangements were made for the congress and exhibition of the Sanitary Institute, to be held at Nottingham in the autumn of this year, the large responsibilities which have been placed upon the country in connection with the war in South Africa, have made it difficult to assure the success of a meeting during the coming year. With the approval, therefore, of H.R.H. the Duke of Cambridge, president, and after consulting with the authorities at Nottingham, it has been decided to postpone the meeting to 1901. H.R.H. the Duke of Cambridge has graciously consented to take the chair at the annual dinner, the date of which has been fixed for Friday, May 11th.

